# Probing the Immunoreceptor Tyrosine-Based Inhibition Motif Interaction Protein Partners with Proteomics

**DOI:** 10.3390/molecules29091977

**Published:** 2024-04-25

**Authors:** Yujun Gao, Shu Xing, Lianghai Hu

**Affiliations:** School of Life Sciences, Jilin University, Changchun 130012, China; yjgao18@jlu.edu.cn

**Keywords:** tyrosine phosphatase, proteomics, mass spectrometry, phosphorylation, ITIM, SH2

## Abstract

Phosphorylation of tyrosine is the basic mode of protein function and signal transduction in organisms. This process is regulated by protein tyrosine kinases (PTKs) and protein tyrosinases (PTPs). Immunoreceptor tyrosine-based inhibition motif (ITIM) has been considered as regulating the PTP activity through the interaction with the partner proteins in the cell signal pathway. The ITIM sequences need to be phosphorylated first to active the downstream signaling proteins. To explore potential regulatory mechanisms, the ITIM sequences of two transmembrane immunoglobulin proteins, myelin P0 protein-related protein (PZR) and programmed death 1 (PD-1), were analyzed to investigate their interaction with proteins involved in regulatory pathways. We discovered that phosphorylated ITIM sequences can selectively interact with the tyrosine phosphatase SHP2. Specifically, PZR-N-ITIM (pY) may be critical in the interaction between the ITIM and SH2 domains of SHP2, while PD1-C-ITSM (pY) may play a key role in the interaction between the ITIM and SH2 domains of SHP2. Quite a few proteins were identified containing the SH2 domain, exhibiting phosphorylation-mediated interaction with PZR-ITIM. In this study, 14 proteins with SH2 structural domains were identified by GO analysis on 339 proteins associated to the affinity pull-down of PZR-N-ITIM (pY). Through the SH2 domains, these proteins may interact with PZR-ITIM in a phosphorylation-dependent manner.

## 1. Introduction

The signaling mechanism responsible for tyrosine phosphorylation is critical for numerous cell processes, including differentiation, proliferation, energy storage, motility, and apoptosis, all of which are closely linked to a range of diseases [1]. Tyrosine phosphorylation has the ability to modulate enzyme activity, regulate protein localization, and orchestrate signal complex assembly, thereby exerting control over downstream cellular events [2]. Two distinct enzyme types balance tyrosine phosphorylation: protein–tyrosine kinases (PTKs), responsible for catalyzing phosphorylation reactions, and protein–tyrosine phosphatases (PTPs) [3,4], which execute the dephosphorylation process [5,6]. Elucidating the functions of these enzymes and identifying their intracellular targets are crucial objectives in current signal transduction research.

The immune system utilizes negative regulators, specifically receptors that contain one or more immunoreceptor tyrosine-based inhibition motifs (ITIMs), which follow the consensus sequence of S/I/V/LxYxxI/V/L [7]. When these inhibitory receptors are bound with ligands, they trigger the phosphorylation of ITIMs by Src kinases and subsequently recruit phosphotyrosine phosphatases (PTPs), including SHP1 (Src Homology 2 Domain-Containing Phosphatase 1) and SHP2 (Src Homology 2 Domain-Containing Phosphatase 2) [8]. We examined the relationships between the ITIMs of two model proteins and their corresponding binding partners in order to obtain an in-depth understanding of how ITIM sequences influence the related downstream pathways. Protein zero related (PZR) is an immunoglobulin superfamily cell surface protein that features a pair of immunoreceptor tyrosine-based inhibitory motifs. Its extracellular segment exhibits a significant sequence homology to myelin P0, a crucial structural component of the myelin sheath [9]. Within its intracellular domain, two ITIMs are present [10], specifically interacting with SHP2 [11], a tyrosine phosphatase containing an SH2 domain that plays a pivotal role in cell signaling [12]. Programmed Death 1 (PD-1), which was initially proposed in 1992, belongs to the CD28 immunoglobulin superfamily [13]. It was originally isolated from an apoptotic mouse T-cell hybridoma, 2B4. PD-1 is a type I transmembrane glycoprotein with a molecular weight of 55 kDa, that consists of three domains: transmembrane, cytoplasmic, and IgG V. Notably, the ITIM and ITSM within the cytoplasmic tail region are crucial for PD-1’s immunosuppressive function. Upon binding to PD-L1, PD-1 recruits the tyrosine phosphatase SHP2 through ITSM, subsequently dephosphorylating numerous key components of the TCR signaling pathway [14,15]. This process inhibits the proliferation and activity of CD4 and CD8 T cells, thereby negatively regulating the immune response [16,17]. The protein phosphatase of SHP2 is essential to the Ras-ERK1/2 signaling pathway [18,19,20,21]. It comprises a multidomain structure, encompassing two adjacent SH2 domains (with N-SH2 designating the N-terminal domain and C-SH2 the C-terminal domain) and a phosphatase domain, organized in the sequence of N-SH2, C-SH2, and phosphatase. SHP2 exhibits allosteric enzyme characteristics [22,23]; specifically, distinct molecular surfaces within the N-SH2 domain are responsible for recognizing phosphotyrosine and the phosphatase domain [24]. There have been reports that both PZR and PD1 interact with SHP2 [25]. However, the preferential binding of SHP2’s SH2 domain to the N-terminal ITIM (N-ITIM) or C-terminal ITIM (C-ITIM) by Tyr-phosphorylated peptides is still not fully understood. Elucidating the nuances of this interaction is critical for gaining a comprehensive understanding of the signaling pathways regulated by these proteins.

To fill this information gap, we explored phosphorylation effects on the interaction between ITIMs and SHP2’s SH2 domain. This was achieved by synthesizing both phosphorylated and non-phosphorylated ITIM peptides derived from PZR and PD1, followed by analysis using Western blot and high-throughput proteomic techniques. The approach allowed us to detect and quantify the specific interactions between these proteins under controlled experimental conditions. By using Western blot, we were able to confirm the binding of SHP2 to phosphorylated ITIM peptides derived from PZR and PD1. Based on the findings, SHP2 demonstrates a preference for binding to phosphorylated ITIMs, consistent with its established role as a phosphotyrosine-binding protein. To accurately determine SHP2’s binding affinity and kinetics towards N-ITIM versus C-ITIM Tyr-phosphorylated peptides, further investigation is warranted. Additionally, we utilized mass spectrometry-based proteomics to identify previously unknown proteins that specifically interact with PZR and its phosphorylated ITIM peptides, aiming to discover novel proteins associated with the PZR-ITIM-SHP2 pathway. The preliminary data revealed that quite a few proteins were potentially associated with the PZR-SHP2 pathway, providing valuable insights into the molecular mechanisms underlying this signaling axis. Taken together, our findings contributed to a deeper understanding of the phosphorylation-dependent interactions between ITIMs and SHP2’s SH2 domain. Future research will focus on understanding the functional implications of these interactions and how they influence the downstream signaling processes in immune cells. Furthermore, identifying proteins involved in the PZR-ITIM-SHP2 pathway may lead to the discovery of new therapeutic targets for modifying immune responses in a variety of disease situations.

## 2. Results and Discussion

### 2.1. Workflow

Phosphorylated ITIMs are recognized for their ability to interact with SH2 domains within proteins, which can trigger downstream signaling events [26]. Tyr241 and Tyr263 embedded in the ITIMs are responsible for the tyrosine phosphorylation of PZR, providing docking sites for the enzyme, for example, responsible for the binding of SHP2. The PD1 intracellular domain comprises an ITIM and an ITSM. ITSM plays an important role in the activation of PD1 because it recruits the protein tyrosine phosphatases SHP1 and SHP2, thereby activating downstream reactions and exerting inhibitory effects [27].

**Figure 1:**
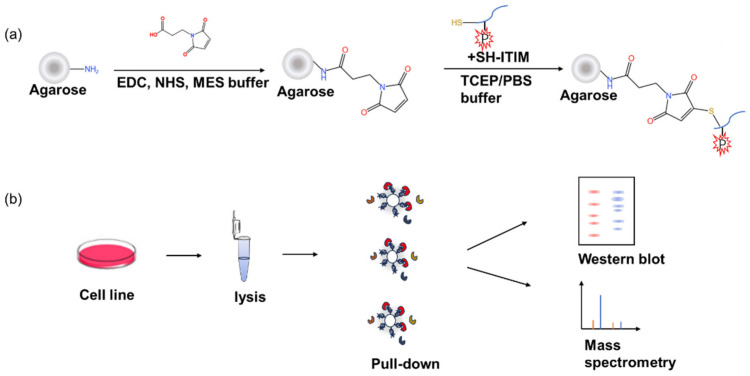
Workflow of the synthesis of agarose beads with different peptides and pull-down experiments: (**a**) synthesis of the ITIM–agarose affinity beads; (**b**) cell lysates were used for affinity pull down by four ITIM–agarose affinity beads, combined with Western blot and proteomic analysis.

To reveal the phosphorylation-mediated interaction between ITIM and SH2, domain synthetic peptides were covalently coupled to agarose beads as an affinity matrix, as shown in Figure 1a. The pull-down complex was then analyzed using Western blot or mass spectrometry-based proteomics for the systematic investigation of the binding partners. The flowchart is shown in Figure 1b. The SPC-A1 cell line was chosen for its importance in a lung cancer-related study [28]. Human non-small cell lung cancer (NSCLC) accounts for more than 80% of all lung cancers [29]. Given that PZR is overexpressed in lung cancer and serves as an unfavorable prognostic biomarker, it has been observed that the depletion of PZR reduces migration, invasion, and ROS levels in SPC-A1 cells in vitro, while suppressing tumor growth in vivo [30]. Human lung adenocarcinoma cell line, SPC-A1(PZR KO) was selected as the research object, which is a type of NSCLC cell with the PZR protein knocked out. When performing PZR peptide affinity pulldown, this cell line was selected because it eliminated the interference of endogenous PZR proteins.

### 2.2. Preparation of Affinity Agarose Beads

To identify novel interacting partners of ITIMs, we generated a peptide containing phosphorylated ITIM or non-phosphorylated ITIM derived from the PZR and PD1, containing a triglycine linker as a spacer and a terminal cysteine residue for convenient immobilization. The peptides PZR-N-ITIM(CGGGGPVIYAQLDHS), PZR-N-ITIM(pY) (CGGGGPVIpYAQLDHS), PZR-C-ITIM(CGGGESVVYADIRKN), and PZR-C-ITIM(pY) (CGGGESVVpYADIRKN), PD1-N-ITIM(CGGGFSVDYGELDFQ), PD1-N-ITIM(pY) (CGGGFSVDpYGELDFQ), PD1-C-ITSM(CGGGEQTEYATIVFP), and PD1-C-ITSM(pY) (CGGGEQTEpYATIVFP) were purchased from the Beijing Zhongke Yaguang Biotechnology Co., Ltd. (Beijing, China) The modified ITIM peptide was immobilized onto agarose resin via a two-step reaction. The surface of agarose beads was modified with maleimide functional groups in the first step. Secondly, the modified ITIM peptides were immobilized via the maleimide–thiol reaction [31]. Finally, the synthesized ITIM–agarose affinity beads were used for subsequent experiments. By comparing the proteins that bind to phosphorylated vs. non-phosphorylated ITIM peptides, it is possible to gain insights into the mechanisms by which ITIM-containing proteins regulate immune responses. ITIMs are known to recruit phosphatases that deactivate signaling molecules involved in immune cell activation, thus serving as a negative regulatory mechanism. Identifying novel ITIM-binding proteins may lead to the discovery of new regulators of immune responses and potential targets for therapeutic intervention.

### 2.3. Affinity Pulldown of the Interaction Partners

**Figure 2:**
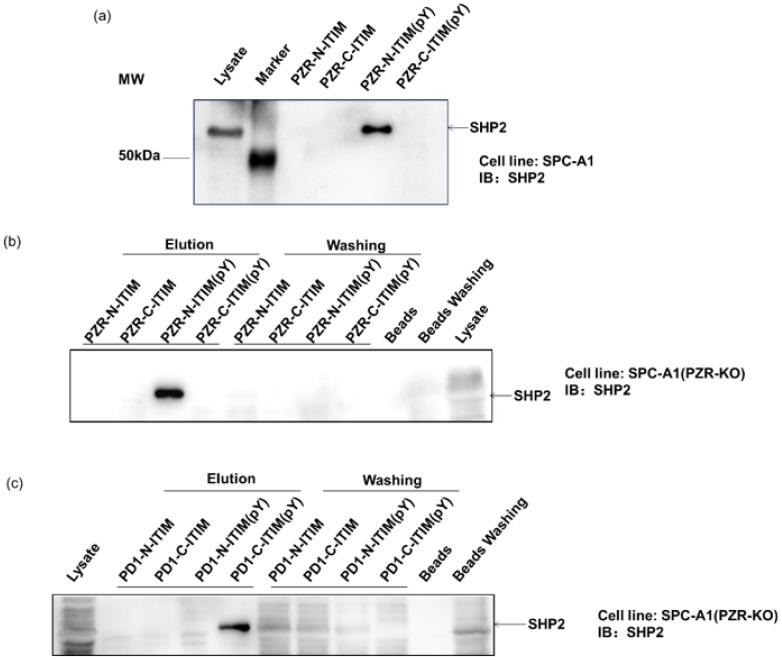
Western blot analysis with SHP2 antibody (1:5000), detected by ECL system. Cell line: SPC-A1. (**a**) Affinity beads with different peptide pull-down SHP2. Cell line: SPC-A1 (**b**) PZR-N-ITIM(pY) pulled down SHP2. Cell line: SPC-A1(PZR KO). (**c**) PD1-C-ITSM(pY) pulled down SHP2. Cell line: SPC-A1(PZR KO).

To investigate the role of individual phosphorylation sites in peptide–protein interactions, a competition binding assay was performed by adding different ITIM-agarose affinity beads into the cell lysates. The protein complex-bound beads were isolated for Western blot analyses to verify their ability to bind proteins, including SHP2. SHP2 is a known interacting protein of PZR and PD1 in human cells, featuring a tandem pair of SH2 domains. This analysis aimed to confirm the specific interactions between the synthetic peptides and SHP2, providing valuable insights into their binding mechanisms in human cells. First, ITIM-agarose affinity beads were synthesized containing different phosphorylation states. These beads were able to mimic the behavior of ITIM motifs in PZR and PD1 proteins, in both phosphorylated and non-phosphorylated states. By mixing these beads with cell lysates, we were able to observe how they interacted with intracellular proteins, particularly SHP2. As shown in Figure 2a, the lysis buffer served as the vehicle control. And after exposure to the SHP2 protein, our synthesized beads were indeed able to pull down SHP2, indicating the successful establishment of this method. Subsequently, we further employed this approach to investigate several ITIM peptide segments individually. During the affinity pull-down experiment, we introduced beads to the cell lysate, thoroughly mixed them, and then separated the beads and supernatant by centrifugation. The possible intercalating proteins of the peptides were then spun down, and the beads were washed off the redundant proteins. In Figure 2b,c, the labeled elution area above the lanes depicts the separated beads, while the labeled washing portion represents the centrifuged supernatant and washing solution. In addition, blank beads were employed as the control. It can be seen from Figure 2b,c that PZR-N-ITIM(pY) can pull down the SHP2 protein from SPC-A1(PZR KO) cell lysates. The other three PZR-ITIM-agarose affinity beads did not pull down the SHP2 protein. In SPC-A1(PZR KO) cell lysates, PD1-C-ITSM(pY) can pull down the SHP2 protein. The other three PD1-ITIM-agarose affinity beads did not pull down the SHP2 protein. Taking into account that SHP2 contains two SH2 domains, which will specifically interact with Tyr-P-peptides, as expected, non-phosphorylated peptides cannot interact with SH2 domains. Thus, PZR-N-ITIM(pY) may play critical roles in the interaction of ITIM and SH2 domains which were allowed to pull down SHP2. PD1-C-ITSM (pY) plays a key role in the interaction between ITIM and SH2 domains, enabling SHP2 to be recruited, as previously reported. The specificity of the ITIM-agarose affinity beads was further confirmed by Western blot analyses, where the supernatant was also analyzed by Western blot, to achieve a better understanding of how the peptide contains two ITIMs interacting with SH2 domains. As shown in Figure 2b,c, both PZR and PD1 were performed with Western blot by centrifuging the beads, which was consistent with the previous results. The supernatant contained less SHP2 compared to lysate. Moreover, blank beads had no adsorption toward SHP2.

### 2.4. Identification of the Unknown Binding Partners by Proteomics

After confirming the method’s ability to isolate interacting proteins, mass spectrometry-based proteomics was employed to comprehensively analyze additional unknown interacting partners on a large scale. On the one hand, it revealed the details of interactions between ITIM and SH2. On the other hand, we investigated whether this method could be used to identify new proteins in a complex sample. ITIM-agarose affinity beads were mixed with the cell lysates and centrifuged for mass spectrometry analyses.

**Figure 3:**
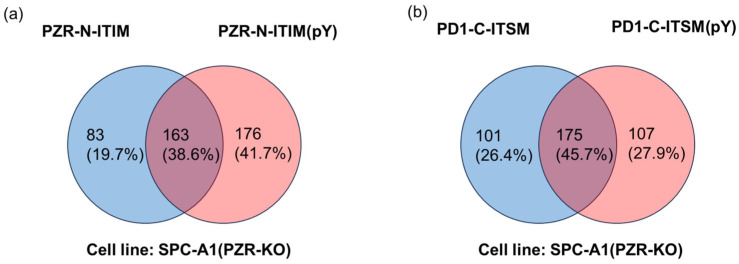
Venn diagram of agarose beads affinity pull-down from SPC-A1(PZR KO) cell line. (**a**) PZR-N-ITIM agarose beads and PZR-N-ITIM(pY) agarose beads affinity pull-down from SPC-A1(PZR KO) cell line. (**b**) PD1-C-ITIM agarose beads and PD1-C-ITIM(pY) agarose beads affinity pull-down from SPC-A1(PZR KO) cell line.

As shown in Figure 3, the pull-down-results showed that PZR-N-ITIM(pY) can pull down 339 proteins and 246 proteins captured by PZR-N-ITIM. It must also be mentioned that both PZR-C-ITIM(pY) and PZR-C-ITIM can capture more proteins, where PZR-C-ITIM(pY) pulled down 699 proteins and 529 proteins were captured by PZR-C-ITIM. It can be speculated that the N-terminal interacts with its interacting proteins more strongly, and the C-terminus can recruit more related proteins. PD1-N-ITIM(pY) pulled down 496 proteins and 345 proteins were captured by PD1-N-ITIM. PD1-C-ITIM(pY) pulled down 276 proteins and 282 proteins were captured by PD1-C-ITIM; more information is depicted in Figure S1. Regardless of whether the peptide segments are phosphorylated or not, proteins that can be pulled down may share structural similarities, enabling them to be recruited and thus contributing to the identification of interacting proteins. However, it is more likely that the phosphorylation signal transduction of ITIMs occurs specifically with proteins pulled down by phosphorylated peptide segments. Therefore, this study focuses on the investigation of ITIMs(pY)–protein interactions. In the proteomic results, it was found that the peptide containing phosphorylated ITIM can pull down SHP2, but other peptides cannot, which is consistent with the results of the Western blot analysis. It not only showed that the results of this proteomic analyses were reliable, but also confirmed that our research method was credible. Therefore, a further detailed analysis was carried out on the results of identified proteins by proteomic analyses. In prior experiments, we successfully identified PZR-N-ITIM(pY) and PD1-C-ITIM(pY) as peptides capable of precipitating SHP2 proteins. Utilizing agarose beads synthesized from these peptides, we subsequently precipitated the proteins from cell lysates. For the purpose of elucidating differences, we conducted mass spectrometry analysis. Detailed analysis was conducted toward the proteomic data to identify potential novel binding partners and to gain further insights into the molecular mechanisms underlying ITIM-mediated protein interactions. This information will be crucial for advancing the understanding of cellular signaling networks and for developing new therapeutic strategies targeting these critical interactions.

### 2.5. Gene Ontology Analysis

**Figure 4:**
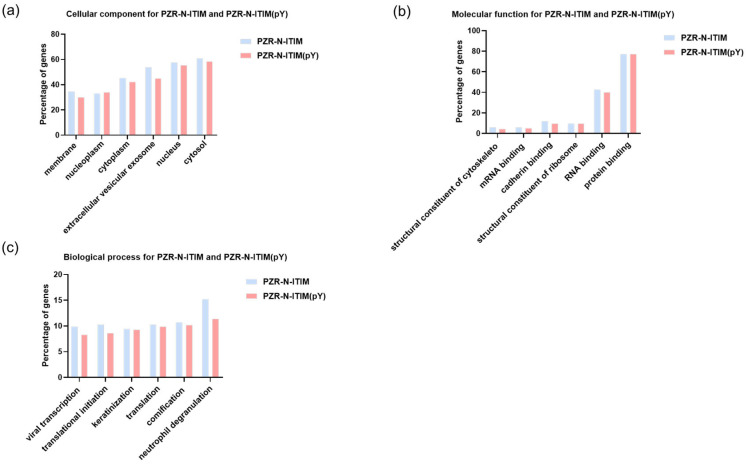
GO analysis of PZR-N-ITIM agarose beads and PZR-N-ITIM(pY) agarose beads affinity pull-down from SPC-A1(PZR KO) cell line. (**a**) Cellular component; (**b**) molecular function; (**c**) biological process.

For both PZR and PD1, the peptides were selected to demonstrate exceptional binding properties along with their respective control peptides. Following this selection, gene ontology (GO) analysis was carried out to predict gene functions and the classification of protein function and expression. This comprehensive classification system encompasses three primary ontologies: molecular function (MF), which focuses on the biochemical activities of gene products; cellular component (CC), which describes the locations of gene products within cells and their larger biological contexts; and biological process (BP), which details the broader biological objectives or outcomes to which gene products contribute [32]. As shown in Figure 4, the results of GO analysis were presented toward the proteins isolated using PZR-ITIM-agarose beads from the SPC-A1(PZR KO) cell line. In Figure 4b, our findings reveal that the number of proteins associated with molecular function is relatively low as compared to the other ontologies. This observation may be attributed to the fact that proteins with molecular functions often constitute a smaller proportion of the total protein pool within a cell. Similarly, the GO analysis of proteins isolated using PD1-ITIM-agarose beads from the SPC-A1(PZR KO) cell line exhibited a comparable trend, as shown in Figure S2, aligning with the findings observed for PZR. Our primary focus was on the outcomes related to PZR. Therefore, the experiments were conducted using PZR knockout cell lines as the main subjects of investigation. To further enhance our understanding of these data, we undertook a more detailed analysis. This comprehensive examination aimed to elucidate the precise roles and interactions of these proteins within the cellular environment, shedding valuable light on their functional significance. By delving deeper into the intricacies of the data, we hope to uncover novel protein interactions and potential regulatory mechanisms that underlie cellular processes. Such insights could pave the way for future research endeavors aimed at elucidating the complex networks that govern cellular function and dysfunction.

### 2.6. PZR–ITIM Interaction Proteins

**Figure 5:**
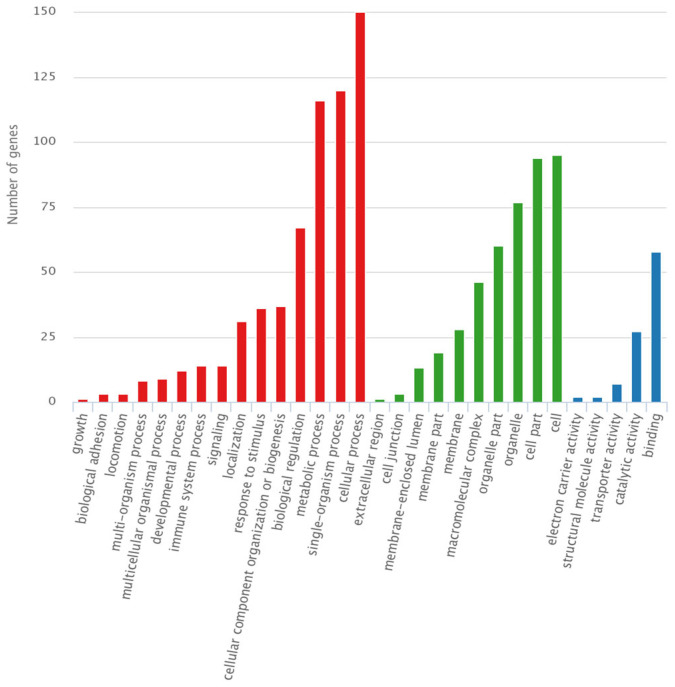
GO analysis of PZR-N-ITIM(pY) agarose beads affinity pull-down from SPC-A1(PZR KO) cell line. Red: biological Process; Green: cellular component; Blue: molecular function.

**Figure 6:**
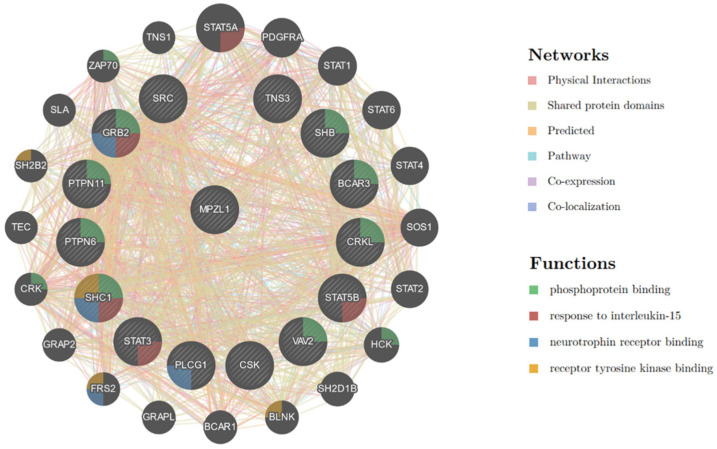
The networks of PZR-N-ITIM(pY) affinity pull-down-proteomic analysis. Each bubble represents a gene corresponding to a protein, and the thickness of the connecting lines between them reflects the tightness of the interconnections between the different proteins.

In addition to previously known proteins (c.a. Src), some new proteins were also identified in this case. Considering that ITIM tends to interact with SH2, the identified proteins that contained the SH2 domain are shown in Table 1. Among them, Src and SHP2 are proteins known to interact with PZR, and others are newly identified proteins. However, there is a close interaction between these proteins, as shown in Figure 5. We further analyzed the protein pulled down by PZR-N-ITIM (PY). From the GO analysis, it can been seen that this peptide is more involved in biological process-related functions. STRING was then used to analyze the relationship between proteins containing SH2. As shown in Figure 6, several proteins were identified, such as proto-oncogene tyrosine-protein kinase Src, non-receptor protein tyrosine kinase which is activated following engagement of many different classes of cellular receptors including immune response receptors, integrins and other adhesion receptors, receptor protein tyrosine kinases, G protein-coupled receptors, as well as cytokine receptors that participate in signaling pathways that control a diverse spectrum of biological activities including gene transcription, immune response, cell adhesion, cell cycle progression, apoptosis, migration, and transformation.

Figure 6 further sketches these relationships in more detail. Among these proteins, STAT3 and GRB2 are known to interact with SHP2 and GRB2 is reported to bind to both PZR and SHP2. Therefore, it is not excluded that STAT3 can directly interact with PZR and requires further verification. At the same time, STAT3 has also been reported to interact with SRC [33]. Recognizing that PZR regulates downstream signaling by recruiting SHP2, we compared the proteins identified via PZR-N-ITIM with those isolated through immunoprecipitation experiments targeting SHP2, as depicted in Figure S3a. Specifically, we focused on the proteins co-precipitated in both assays, as they were highly likely to be associated with the functional role of PZR within the cellular environment. To further elucidate the function of PZR, we conducted a network analysis on these co-precipitated proteins, as presented in Figure S3b, which provided additional insights into the functional mechanisms of PZR [34,35].

## 3. Materials and Methods

### 3.1. Cell Lines and Cell Culture

The SPC-A1 cells were incubated at 37 °C in an atmosphere containing 5% CO_2_, utilizing Dulbecco’s modified Eagle medium (DMEM) (Gibco, New York, NY, USA) supplemented with 10% (*v*/*v*) fetal bovine serum (FBS) (Gibco, New York, NY, USA), along with 100 units/mL of penicillin and 100 units/mL of streptomycin.

### 3.2. Synthesis of the ITIM-Agarose Affinity Beads

In this study, four PZR ITIM peptides: PZR-N-ITIM(CGGGGPVIYAQLDHS), PZR-N-ITIM(pY) (CGGGGPVIpYAQLDHS), PZR-C-ITIM(CGGGESVVYADIRKN), and PZR-C-ITIM(pY) (CGGGESVVpYADIRKN) and four PD1 ITIM peptides: PD1-N-ITIM(CGGGFSVDYGELDFQ), PD1-N-ITIM(pY) (CGGGFSVDpYGELDFQ), PD1-C-ITSM(CGGGEQTEYATIVFP), and PD1-C-ITSM(pY) (CGGGEQTEpYATIVFP) were used. All the above peptides were purchased from the Beijing Zhongke Yaguang Biotechnology Co., Ltd. A slurry of 1 mL agarose beads (~16 μmol amino groups) was thoroughly washed three times with water. Subsequently, the beads were reacted with 32 μmol of 3-maleimidopropionic acid, 64 μmol of NHS (N-hydroxysulfosuccinimide), and 640 μmol of EDC (1-ethyl-3-(3-dimethylaminopropyl) carbodiimide) in a 2 mL solution of 200 mM MES buffer. This reaction was carried out for 16 h at 4 °C. Afterward, the maleimide-modified beads were reacted with 16 μmol of peptides in a 5 mM solution of tris(2-carboxyethyl) phosphine (TCEP) at room temperature for 30 min. Subsequently, the beads were incubated in a 50 mM cysteine solution for 2 h to block any unreacted maleimide groups. Finally, the modified beads were washed with 1 M NaCl and stored in PBS buffer for further utilization.

### 3.3. Cell Lysis

The cells were washed with phosphate-buffered saline (PBS) prior to lysis. Lysis was achieved in a WCEB buffer, which comprised 50 mM Tris-Cl (pH 7.5), 0.15 M NaCl, 1 mM EDTA, 0.2% Triton X-100, 0.1 M microcystin, 1 mM benzamidine, 0.1 mM phenylmethylsulfonyl fluoride, 20 g/mL leupeptin, 1 M pepstatin, and 1 g/mL aprotinin. The lysates were then centrifuged at 13,000× *g* for 30 min at 4 °C. The protein concentration of the resulting supernatant was determined using the Bradford assay.

### 3.4. Affinity Pull-Down

The ITIM-agarose beads (50 μL slurry) were incubated with 200 μL of cell lysates by rotating overnight at 4 °C. The beads were washed three times with a washing buffer containing 25 mM glycerophosphate (pH 7.5), 0.15 M NaCl, 1 mM EDTA, 1 mM EGTA, 2 mM mercaptoethanol, 0.1% Triton X-100, 0.25 mM Na3VO4, and plus Protease Inhibitors (1.0 mM benzamidine, 0.1 mM phenylmethylsulfonyl fluoride, 20 g/mL leupeptin, 1 M pepstatin, and 1 g/mL aprotinin). The beads were separated by centrifugation and washed three times with PBS for subsequent experiments.

### 3.5. Western Blot Analyses

Samples were resolved using 12% SDS-PAGE and subsequently transferred onto polyvinylidene difluoride (PVDF) membranes with a pore size of 0.45 μm (Millipore, Milford, MA, USA). Blocking was performed with a buffer containing 5% bovine serum albumin (BSA) in TBST (consisting of 0.1% Tween-20, 150 mM NaCl, and 20 mM Tris-HCl at pH 7.5) for 20 min at room temperature. Subsequently, the membranes were incubated overnight at 4 °C with SHP2 antibody diluted 1:5000 in the blocking buffer. Following three washes with TBST, the membranes were incubated at 37 °C for 1.5 h with the relevant HRP-labeled secondary antibody, and also diluted 1:5000 in the blocking buffer. After three additional washes with TBST, the membranes were visualized using the ECL detection method.

### 3.6. Protein Digestion

Prior to mass spectrometry analysis, the pull-down ITIM interaction proteins underwent a digestion process as follows. Initially, 200 μL of denaturant, consisting of 8 M urea in 100 mM NH_4_HCO_3_ and 10 μL of DTT (1 mg/mL), was added to the protein sample. This mixture was then shaken for 2 h at 37 °C to ensure thorough denaturation. Subsequently, 1.4 mg of IAA was added to the samples and incubated in the dark for 40 min to allow for alkylation. This was followed by the addition of seven volumes of a buffer containing 100 mM NH4HCO3 to dilute the denaturant.

After dilution, 20 μL of trypsin (1 mg/mL) was added to the samples and incubated overnight at a stable temperature of 37 °C to obtain tryptic peptides. The resulting samples were then desalted using a 100 mg Sep-Pak C18 column (Waters, Milford, MA, USA), following the manufacturer’s instructions. Finally, the samples were dried in a speed vacuum to remove any residual solvents before undergoing mass spectrometry analysis.

### 3.7. LC-MS/MS Analyses

The dried peptides were reconstituted in a solution containing 15 μL of 0.1% formic acid (FA) and 2% acetonitrile (ACN). For proteomic analysis, liquid chromatography–tandem mass spectrometry (LC-MS/MS) was performed using a Dionex UltiMate 3000 RSL nano system (Thermo Scientific, Waltham, MA, USA) in conjunction with an Orbitrap Fusion Lumos Tribrid spectrometer (Thermo Scientific, USA). The samples were separated on a custom-packed 15 cm column (internal diameter of 150 μm) filled with C18 AQ beads (1.9 μm; Dr. Maisch, GmbH, Ammerbuch, Germany). The mobile phase composition was designed as follows: mobile phase A consisted of 98% water and 2% ACN, while mobile phase B comprised 80% ACN and 20% water; both phases contained 0.1% FA. During the proteomic analysis, a constant flow rate of 600 nL/min was maintained, employing a linear gradient spanning 100 min. Specifically, the elution gradient was initially set to 1% mobile phase B for 10 min, followed by a linear increase from 1% to 34% mobile phase B over 60 min. Subsequently, the gradient rose linearly from 34% to 90% mobile phase B within 15 min. After maintaining 90% mobile phase B for 12 min, the content of mobile phase B was gradually returned to 1% and held steady for 3 min. The mass spectrometer operated in a data-dependent mode, enabling precise proteomic analysis.

### 3.8. Data Processing

Using MaxQuant, RAW files were searched against the UniProt human database (www.uniprot.org, accessed on 4 June 2018.) with the following default search parameter settings: mass tolerance of 10 ppm for precursor ions and 20 ppm for fragment ions; fixed modification of cysteine residues through carbamidomethylation (+57.0214 Da); variable modification of methionine residues through oxidation (+15.9949 Da). The false discovery rates (FDR) for both proteins and peptides were stringently set at 0.01 to ensure the accuracy and reliability of the results.

## 4. Conclusions

In this study, ITIM-agarose affinity beads were synthesized through a two-step reaction using peptides comprising either phosphorylated or non-phosphorylated ITIM sequences derived from PZR and PD1. The affinity beads were successfully used for Western blot validation of the known interaction proteins. More importantly, large scale identification of unknown binding partners was revealed by the combination of mass spectrometry-based proteomics. Our studies suggest that PZR-N-ITIM(pY) may play a pivotal role in mediating the interaction between the ITIM domain and the SH2 domain of SHP2. Similarly, PD1-C-ITSM(pY) is believed to be a key player in facilitating the interaction between ITIM and the SH2 domain of SHP2. Using the PZR-N-ITIM(pY) peptide, we were able to pull down and identify a number of proteins that contained SH2 domains. These proteins are known to engage in phosphorylation-mediated interactions with the PZR-ITIM, highlighting the potential importance of this pathway in cellular signaling and regulation. Our findings may contribute to a growing understanding of the complex network of protein–protein interactions that underlie cellular function and may lead to the discovery of new therapeutic targets.

## Figures and Tables

**Table 1:** A partial list of proteins that contained the SH2 domain identified in PZR-N-ITIM(pY) affinity pull-down. Results of SPC-A1(PZR KO) cell line.

UniProt	Protein Name
P46109	CRK-like proto-oncogene, adaptor protein (CRKL)
Q15464	SH2 domain containing adaptor protein B (SHB)
P29353	SHC adaptor protein 1 (SHC1)
P12931	SRC proto-oncogene, non-receptor tyrosine kinase (SRC)
O75815	Breast cancer anti-estrogen resistance 3 (BCAR3)
P41240	c-src tyrosine kinase (CSK)
P62993	Growth factor receptor-bound protein 2 (GRB2)
P19174	Phospholipase C gamma 1 (PLCG1)
Q06124	Protein tyrosine phosphatase, non-receptor type 11 (PTPN11)
P29350	Protein tyrosine phosphatase, non-receptor type 6 (PTPN6)
P40763	Signal transducer and activator of transcription 3 (STAT3)
P51692	Signal transducer and activator of transcription 5B (STAT5B)
Q68CZ2	tensin 3 (TNS3)
P52735	VAV guanine nucleotide exchange factor 2 (VAV2)

## Data Availability

Data are contained within the article.

## Supplementary Materials

The following supporting information can be downloaded at: https://www.mdpi.com/article/10.3390/molecules29091977/s1, Figure S1: result of ITIM–agarose beads affinity pull-down from SPC-A1(PZR KO) cell line. Venn diagram showing the overlap of proteins from each affinity pull-down-MS: (a) the Venn diagram of the proteins pulled down by PZR-ITIM agarose beads, with the number of proteins and their proportion labeled within the diagram; (b) the Venn diagram of the proteins pulled down by PD1-ITIM agarose beads, with the number of proteins and their proportion labeled within the diagram; Figure S2: GO analysis of PD1-C-ITSM agarose beads and PZR-C-ITSM(pY) agarose beads affinity pull-down from SPC-A1(PZR KO) cell line: (a) cellular component; (b) molecular function; (c) biological process; Figure S3: comparison of PZR-N-ITIM(pY) and SHP2 proteins pulled down in the SPC-A1 cell line: (a) the overlap of proteins from PZR-N-ITIM(pY) affinity pull-down and SHP2 IP for MS; (b) the networks of PZR-N-ITIM(pY) affinity pull-down and SHP2 IP for MS analysis; Figure S4. Uncropped Western blot analysis with SHP2 antibody (1:5000), detected by ECL system. Cell line: SPC-A1. (a) Affinity beads with different peptide pull-down SHP2. (b) PZR-N-ITIM (pY) pulled down SHP2. (c) PD1-C-ITSM(pY) pulled down SHP2; Tables S1–S7: protein list for the identification of affinity pull-down by peptides and SHP2 conjugated beads.

## References

[1] Frankson R., Yu Z.H., Bai Y.P., Li Q.L., Zhang R.Y., Zhang Z.Y. (2017). Therapeutic Targeting of Oncogenic Tyrosine Phosphatases. Cancer Res..

[2] Östman A., Hellberg C., Böhmer F.D. (2006). Protein-tyrosine phosphatases and cancer. Nat. Rev. Cancer.

[3] Alonso A., Sasin J., Bottini N., Friedberg I., Friedberg I., Osterman A., Godzik A., Hunter T., Dixon J., Mustelin T. (2004). Protein tyrosine phosphatases in the human genome. Cell.

[4] Tautz L., Critton D.A., Grotegut S. (2013). Protein tyrosine phosphatases: Structure, function, and implication in human disease. Methods Mol. Biol..

[5] Tonks N.K. (2006). Protein tyrosine phosphatases: From genes, to function, to disease. Nat. Rev. Mol. Cell Biol..

[6] Van Huijsduijnen R.H., Bombrun A., Swinnen D. (2002). Selecting protein tyrosine phosphatases as drug targets. Drug Discov. Today.

[7] Barrow A.D., Trowsdale J. (2006). You say ITAM and I say ITIM, let’s call the whole thing off: The ambiguity of immunoreceptor signalling. Eur. J. Immunol..

[8] Xu X.Z., Masubuchi T., Cai Q.X., Zhao Y., Hui E.F. (2021). Molecular features underlying differential SHP1/SHP2 binding of immune checkpoint receptors. eLife.

[9] Raasakka A., Kursula P. (2020). How Does Protein Zero Assemble Compact Myelin?. Cells.

[10] Roubelakis M.G., Tsaknakis G., Lyu F.J., Trohatou O., Zannettino A.C.W., Watt S.M. (2020). P_0_-Related Protein Accelerates Human Mesenchymal Stromal Cell Migration by Modulating VLA-5 Interactions with Fibronectin. Cells.

[11] Zannettino A.C.W., Roubelakis M., Welldon K.J., Jackson D.E., Simmons P.J., Bendall L.J., Henniker A., Harrison K.L., Niutta S., Bradstock K.F. (2003). Novel mesenchymal and haematopoietic cell isoforms of the SHP-2 docking receptor, PZR: Identification, molecular cloning and effects on cell migration. Biochem. J..

[12] Zhao Z.J., Zhao R. (1998). Purification and cloning of PZR, a binding protein and putative physiological substrate of tyrosine phosphatase SHP-2. J. Biol. Chem..

[13] Hui E.F., Cheung J., Zhu J., Su X.L., Taylor M.J., Wallweber H.A., Sasmal D.K., Huang J., Kim J.M., Mellman I. (2017). T cell costimulatory receptor CD28 is a primary target for PD-1-mediated inhibition. Science.

[14] Li H.F., Seeram N.P., Liu C., Ma H. (2022). Further investigation of blockade effects and binding affinities of selected natural compounds to immune checkpoint PD-1/PD-L1. Front. Oncol..

[15] Kleffel S., Posch C., Barthel S.R., Mueller H., Schlapbach C., Guenova E., Elco C.P., Lee N., Juneja V.R., Zhan Q. (2015). Melanoma Cell-Intrinsic PD-1 Receptor Functions Promote Tumor Growth. Cell.

[16] Brown K.E., Freeman G.J., Wherry E.J., Sharpe A.H. (2010). Role of PD-1 in regulating acute infections. Curr. Opin. Immunol..

[17] Liu X.N., Zhao A., Xiao S., Li H.H., Li M.H., Guo W., Han Q.J. (2024). PD-1: A critical player and target for immune normalization. Immunology.

[18] Bentires-Alj M., Paez J.G., David F.S., Keilhack H., Halmos B., Naoki K., Maris J.M., Richardson A., Bardelli A., Sugarbaker D.J. (2004). Activating mutations of the Noonan syndrome-associated *SHP2*/*PTPN11* gene in human solid tumors and adult acute myelogenous leukemia. Cancer Res..

[19] Cheng Y.P., Chiu H.Y., Hsiao T.L., Hsiao C.H., Lin C.C., Liao Y.H. (2013). Scalp melanoma in a woman with LEOPARD syndrome: Possible implication of PTPN11 signaling in melanoma pathogenesis. J. Am. Acad. Dermatol..

[20] Kusano K.I., Thomas T.N., Fujiwara K. (2008). Phosphorylation and localization of protein-zero related (PZR) in cultured endothelial cells. Endothelium.

[21] Van Vliet C., Bukczynska P.E., Puryer M.A., Sadek C.M., Shields B.J., Tremblay M.L., Tiganis T. (2005). Selective regulation of tumor necrosis factor-induced Erk signaling by Src family kinases and the T cell protein tyrosine phosphatase. Nat. Immunol..

[22] Hof P., Pluskey S., Dhe-Paganon S., Eck M.J., Shoelson S.E. (1998). Crystal structure of the tyrosine phosphatase SHP-2. Cell.

[23] Chen Y.N.P., LaMarche M.J., Chan H.M., Fekkes P., Garcia-Fortanet J., Acker M.G., Antonakos B., Chen C.H.T., Chen Z.L., Cooke V.G. (2016). Allosteric inhibition of SHP2 phosphatase inhibits cancers driven by receptor tyrosine kinases. Nature.

[24] Neel B.G., Gu H.H., Pao L. (2003). The ‘Shp’ing news: SH2 domain-containing tyrosine phosphatases in cell signaling. Trends Biochem. Sci..

[25] Barford D., Neel B.G. (1998). Revealing mechanisms for SH2 domain mediated regulation of the protein tyrosine phosphatase SHP-2. Structure.

[26] Cheng Y., Ouyang W.W., Liu L., Tang L.K., Zhang Z.G., Yue X.R., Liang L., Hu J.P., Luo T. (2024). Molecular recognition of ITIM/ITSM domains with SHP2 and their allosteric effect. Phys. Chem. Chem. Phys..

[27] Marasco M., Berteotti A., Weyershaeuser J., Thorausch N., Sikorska J., Krausze J., Brandt H.J., Kirkpatrick J., Rios P., Schamel W.W. (2020). Molecular mechanism of SHP2 activation by PD-1 stimulation. Sci. Adv..

[28] Chai X., Zhang X.R., Li W.Q., Chai J. (2021). Small cell lung cancer transformation during antitumor therapies: A systematic review. Open Med..

[29] Ganti A.K., Klein A.B., Cotarla I., Seal B., Chou E. (2021). Update of Incidence, Prevalence, Survival, and Initial Treatment in Patients With Non-Small Cell Lung Cancer in the US. JAMA Oncol..

[30] Fu Y., Sui Y., Zhao Y.M., Jiang J.Z., Wang X.Y., Cui J.R., Fu X.Q., Xing S., Zhao Z.J. (2023). PZR promotes tumorigenicity of lung cancer cells by regulating cell migration and invasion via modulating oxidative stress and cell adhesion. Aging.

[31] Hu L.H., Yang L., Lipchik A.M., Geahlen R.L., Parker L.L., Tao W.A. (2013). A Quantitative Proteomics-Based Competition Binding Assay to Characterize pITAM-Protein Interactions. Anal. Chem..

[32] Blake J.A., Dolan M., Drabkin H., Hill D.P., Ni L., Sitnikov D., Bridges S., Burgess S., Buza T., McCarthy F. (2013). Gene Ontology Annotations and Resources. Nucleic Acids Res..

[33] Akira S. (1999). Functional roles of STAT family proteins: Lessons from knockout mice. Stem Cells.

[34] Yi J.S., Perla S., Enyenihi L., Bennett A.M. (2020). Tyrosyl phosphorylation of PZR promotes hypertrophic cardiomyopathy in *PTPN11*-associated Noonan syndrome with multiple lentigines. JCI Insight.

[35] Zehender A., Huang J.G., Györfi A.H., Matei A.E., Thuong T.M., Xu X.H., Li Y.N., Chen C.W., Lin J.P., Dees C. (2018). The tyrosine phosphatase SHP2 controls TGFβ-induced STAT3 signaling to regulate fibroblast activation and fibrosis. Nat. Commun..

