# Supplementary material for: Probing the Immunoreceptor Tyrosine-Based Inhibition Motif Interaction Protein Partners with Proteomics
Source: Molecules. 2024 Apr 25;29(9):1977. doi: 10.3390/molecules29091977 (PMC11085718; doi:10.3390/molecules29091977)
Supplement: Supplementary file 1 [file molecules-29-01977-s001.zip › Figure S4-Uncropped WB.pdf]

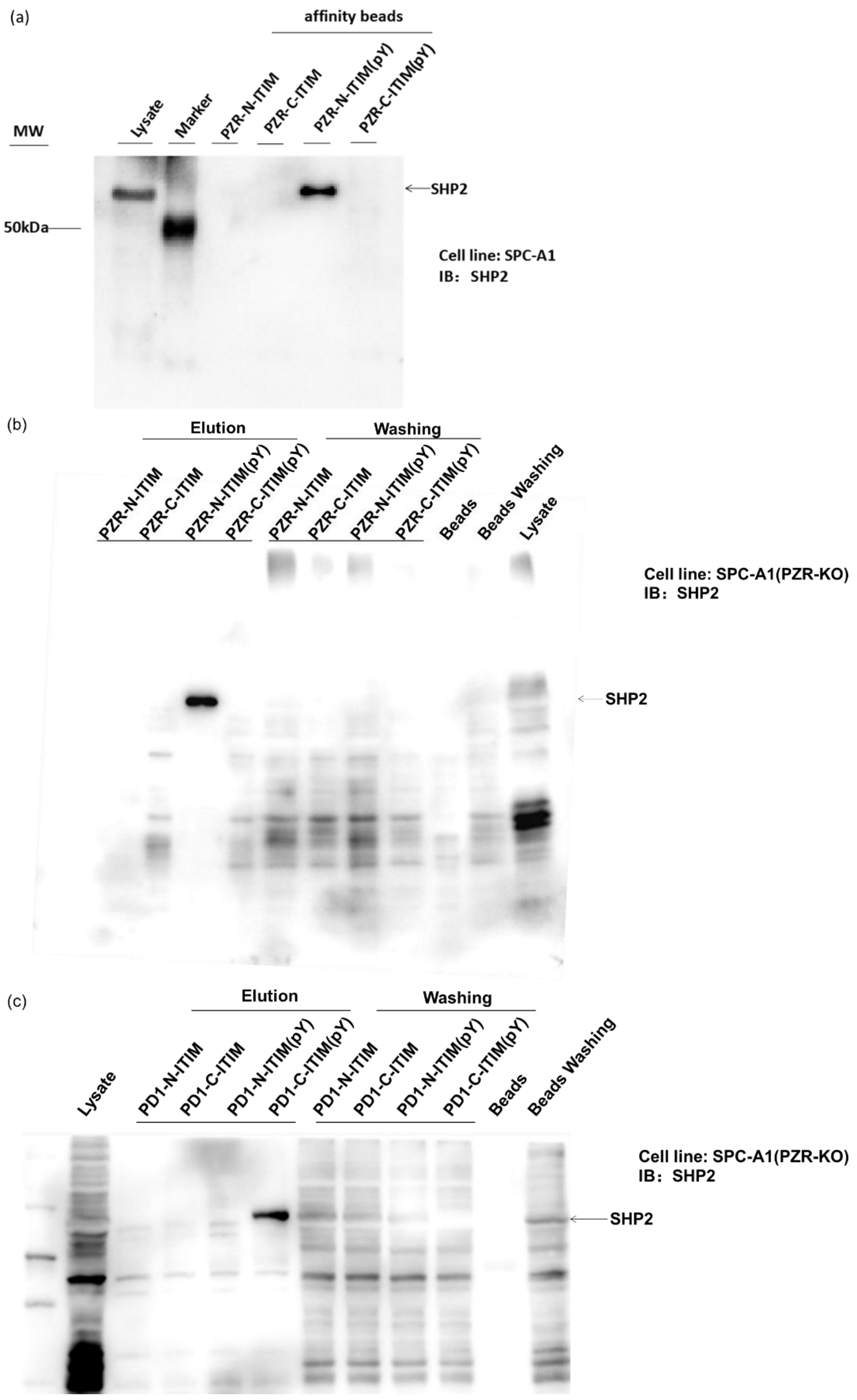

Figure S4. Uncropped Western blot analysis with SHP2 antibody (1:5000), detected by ECL system. Cell line: SPC-A1. (a) Affinity beads with different peptide pull-down SHP2. (b) PZR-N-ITIM (pY) pulled down SHP2. (c) PD1-C-ITSM(pY) pulled down SHP2.
