# Supplementary material for: Probing the Immunoreceptor Tyrosine-Based Inhibition Motif Interaction Protein Partners with Proteomics
Source: Molecules. 2024 Apr 25;29(9):1977. doi: 10.3390/molecules29091977 (PMC11085718; doi:10.3390/molecules29091977)
Supplement: Supplementary file 1 [file molecules-29-01977-s001.zip › Figures S1-S3.pdf]

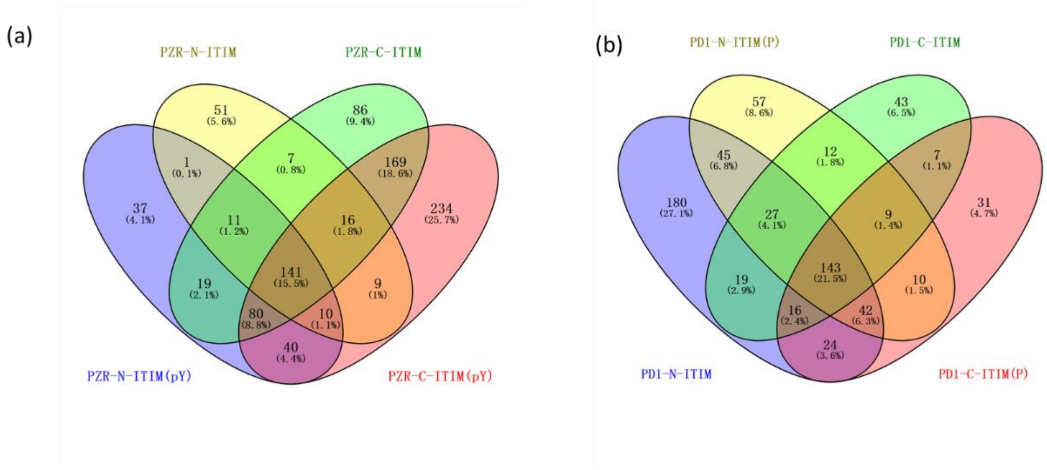

Figure S1. Results of ITIM–agarose beads affinity pull-down from SPC-A1(PZR KO) cell line. Overlap of proteins from each affinity pull-down experiments. (a) The Venn diagram of the proteins pulled down by PZR-ITIM agarose beads, with the number of proteins and their proportion labeled within the diagram. (b) The Venn diagram of the proteins pulled down by PD1-ITIM agarose beads, with the number of proteins and their proportion labeled within the diagram.

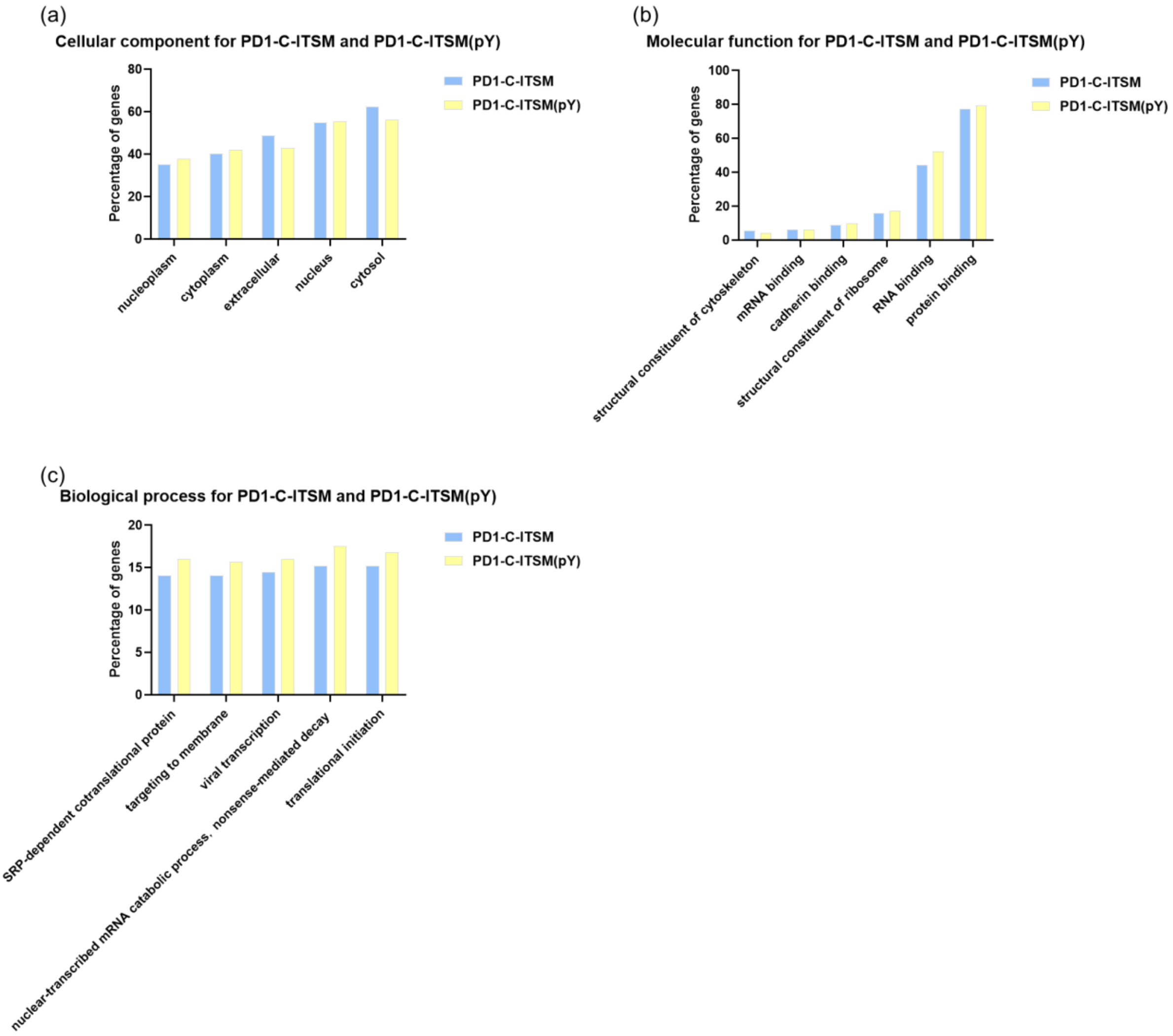

Figure S2. GO analysis of PD1-C-ITSM agarose beads and PZR-C-ITSM(pY) agarose beads affinity pull-down from SPC-A1(PZR KO) Cell Line. (a) Cellular component. (b) Molecular function. (c) Biological process.

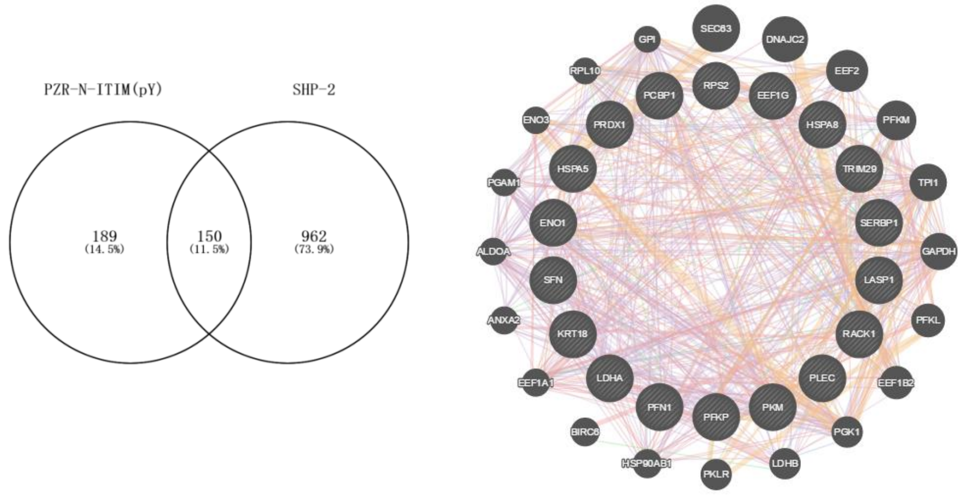

Figure S3. Comparison of PZR-N-ITIM(pY) and SHP2 proteins pulled down in the SPC-A1 cell line (a)The overlap of proteins from PZR-N-ITIM(pY) Affinity Pull-Down for MS and SHP2 IP for MS. (b)The networks of PZR-N-ITIM(pY) Affinity Pull-Down-MS and SHP2 IP for MS analysis.
